# Fault Detection and Diagnosis of Railway Point Machines by Sound Analysis

**DOI:** 10.3390/s16040549

**Published:** 2016-04-16

**Authors:** Jonguk Lee, Heesu Choi, Daihee Park, Yongwha Chung, Hee-Young Kim, Sukhan Yoon

**Affiliations:** 1 Department of Computer and Information Science, Korea University, Sejong Campus, Sejong City 30019, Korea; eastwest9@korea.ac.kr (J.L.); chlgmltn420@korea.ac.kr (H.C.); ychungy@korea.ac.kr (Y.C.); 2 Department of Applied Statistics, Korea University, Sejong Campus, Sejong City 30019, Korea; starkim@korea.ac.kr; 3 Sehwa R&D Center, Techno 2-ro, Yuseong-gu, Daejeon 34026, Korea; shy5406@sehwa.biz

**Keywords:** railway point machine, railway condition monitoring system, audio data, support vector machine

## Abstract

Railway point devices act as actuators that provide different routes to trains by driving switchblades from the current position to the opposite one. Point failure can significantly affect railway operations, with potentially disastrous consequences. Therefore, early detection of anomalies is critical for monitoring and managing the condition of rail infrastructure. We present a data mining solution that utilizes audio data to efficiently detect and diagnose faults in railway condition monitoring systems. The system enables extracting mel-frequency cepstrum coefficients (MFCCs) from audio data with reduced feature dimensions using attribute subset selection, and employs support vector machines (SVMs) for early detection and classification of anomalies. Experimental results show that the system enables cost-effective detection and diagnosis of faults using a cheap microphone, with accuracy exceeding 94.1% whether used alone or in combination with other known methods.

## 1. Introduction

Railway points provide different routes to trains, by driving switchblades between various predetermined positions. The failure of railway points can significantly affect train operations [1]. Consequently, early detection of anomalies is critical for managing railway condition monitoring systems. Technologies for collecting and analyzing data from railway point machinery should be developed in order to minimize detrimental effects of point failure.

Many railway condition monitoring systems are equipped with alarms that apply thresholds to electrical sensor readings. However, the application of thresholds does not ensure early detection of faults [2]. In addition to techniques based on electrical thresholds, the literature includes a wide variety of methods for detecting faults in railway points, including statistical analysis, classification, and model-based methods [3,4,5,6,7]. In particular, classification methods are widely used for detecting faults in a variety of point machinery [2].

Several recent studies reported on SVM-based classification methods [8,9,10] using electrical signals. Eker *et al.* [11] detected faults using principal component analysis together with SVM based on the measurements of a linear ruler, and classified 20 railway point system operations as either fault-free or indicative of drive misalignment. Asada *et al.* [1] showed that current and voltage sensors can be used to collect electrical active power data for railway condition monitoring systems. They reported that the combined use of wavelet transforms and SVMs enabled quite accurate detection and diagnosis of misalignment faults in electrical railway point machinery. Vileiniskis *et al.* [2] presented a methodology for early warning of possible point failure through early detection of changes in the current drawn by the point motor, which was more accurate than commonly used threshold-based methods. Although there has been some recent progress in monitoring railway point systems using electrical signals such as current and voltage, to the best of our knowledge no previous studies have employed audio sensors for automated investigation of anomalies.

Unlike other current research approaches, this paper puts forth a data mining solution that employs audio data to detect faults in railway condition monitoring systems. Firstly, in the data-preprocessing phase, MFCC is extracted and feature dimensions are reduced. Two SVMs are used to detect and diagnose fault sounds, respectively. Experimental results show that this method enables cost-effective detection and diagnosis of faults achieving high accuracy levels of 94.1% for detection, and 97.0% for diagnosis using a cheap microphone. This is the first study on the detection and diagnosis of faults in railway condition monitoring systems via audio data. The results indicate that acoustic analysis of railway sounds can be a reliable method for understanding the condition of railway point machinery. The remainder of this article is structure as follows: Section 2 describes the proposed fault detection and diagnosis of railway point machines, Section 3 presents the results of simulations, and Section 4 draws the conclusions.

## 2. Fault Detection and Diagnosis of Railway Point Machines by Audio Analysis

**Figure 1:**
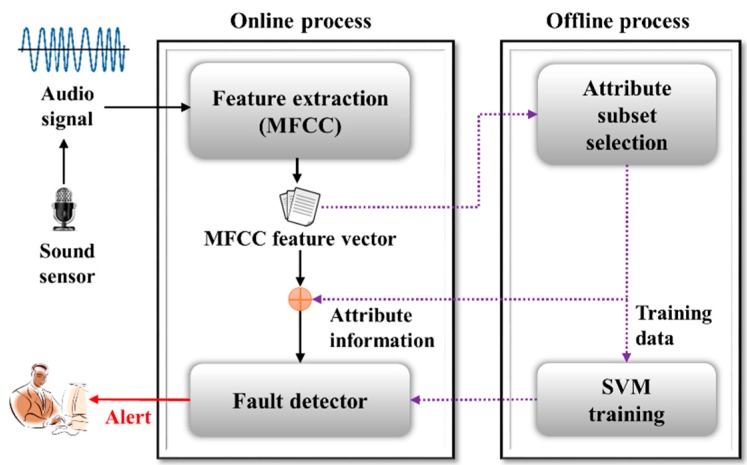
Overall structure for proposed fault detection and diagnosis system by audio analysis.

**Figure 2:**
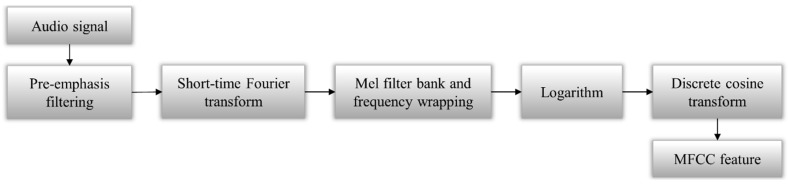
MFCC extraction procedure [17].

The proposed real-time system consists of four modules: two online process modules consisting of a feature extraction module and a fault detector module, and two offline process modules consisting of an attribute subset-selection module and an SVM training module (refer to Figure 1). The feature extraction module is based on the MFCC algorithm [12,13,14,15,16,17] (refer to Figure 2).

**Figure 3:**
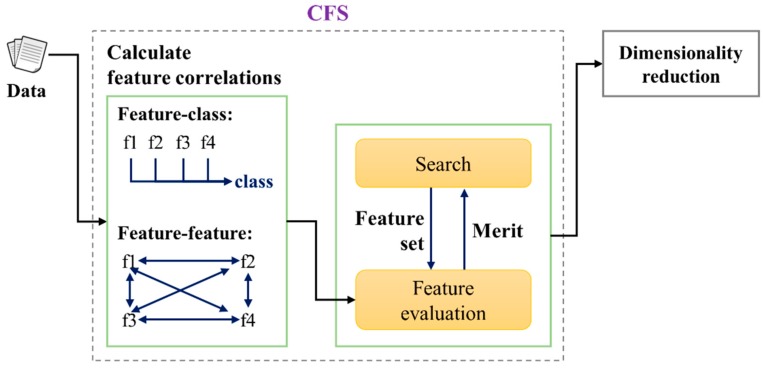
Feature dimensionality reduction by CFS [20].

The attribute subset-selection module is used to select the optimal feature subset with a view to improving the detection and classification speed of the entire diagnosis system. This study uses correlation-based feature selection (CFS), which is one of the most popular attribute subset-selection methods [18,19,20,21] (Figure 3). Following training, the fault detection and classification module detects fault sounds by identifying incoming audio signals and classifying them as subsidiary fault-sound types such as “ice obstruction”, “ballast obstruction”, or “slackened nut”. Although the SVM training module is intended to perform training offline based on the MFCC and CFS, the process is not necessary during the online process.

### 2.1. Mel-frequency Cepstrum Coefficients

The main purpose of feature extraction is to obtain the sequence of feature vectors, thereby providing a compact representation from the raw input signal [12]. Sound analysis research has investigated various acoustic features for use in signal analysis, such as perceptual linear prediction (PLP) features, linear prediction cepstral coefficients (LPCC), and MFCC. In particular, MFCC is widely used in automatic speech recognition and audio analysis, with its simple processing, outstanding ability, containing both time and frequency information, and other advantages [12,13,14,15,16,17]. Additionally, MFCC has been successfully applied to fault diagnosis of engines [22], early classifications of bearing faults [23], and quality assurance of sound signaling devices [24]. Figure 2 shows a structure diagram for MFCC extraction. Firstly, pre-emphasis filtering is used to spectrally flatten the signal. Secondly, the short-time Fourier transform (STFT) is applied to extract information on time and frequency from the input signal. In the mel-frequency wrapping step, the frequency is changed from Hz to mel scale. Then, the mel-frequency signal is converted by the logarithmic mel-spectrum back to the time domain. Finally, discrete cosine transform (DCT) is applied to the log-mel-frequency.

### 2.2. Correlation-Based Feature Selection

The literature includes a wide variety of feature-selection methods, including CFS, gain ratio (GR), principal component analysis (PCA), *etc.* This study uses CFS, which is one of the most popular attribute subset-selection methods [18,19,20,21]. The main objective of CFS is to obtain the highly relevant subset of features, which are uncorrelated to each other [18,19,20,21]. In this way, the dimensionality of data sets can be drastically reduced and the performance of learning algorithms can be maintained or improved. CFS employs heuristic evaluation of the worth or merit of a subset of features. The merit function considers the usability of individual features for predicting the class label, along with the level of inter-correlation among them [18,19,20,21] (refer to Equation (1)). Figure 3 shows a structure diagram of CFS processing. Firstly, feature correlations between feature–class and feature–feature are calculated using symmetrical uncertainty, and then search the feature subset space. After estimating symmetrical uncertainty, the merit of a subset is calculated to find the subset with the highest-ranked merit value:

(1) $$Merit_{F}=\frac{n\bar{r_{cf}}}{\sqrt{n+n\left(n-1\right)\bar{r_{ff}}}}$$

In Equation (1), a feature subset *F* contains n features; $\bar{r_{cf}}$ and $\bar{r_{ff}}$ represent average feature–class correlation and average feature–feature correlation, respectively.

### 2.3. Support Vector Machine

**Figure 4:**
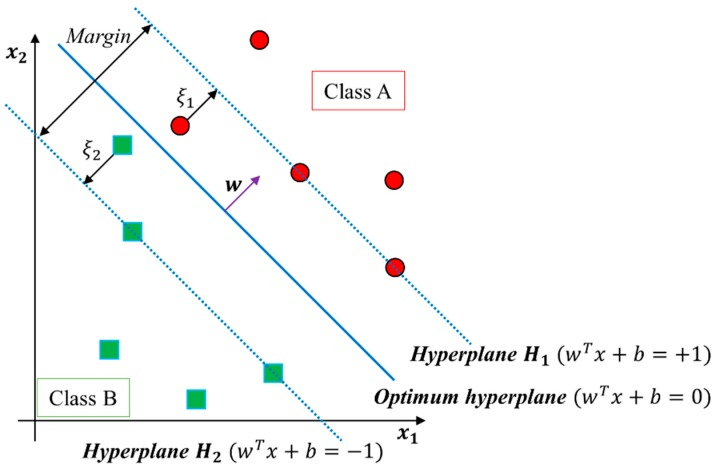
Main concept of SVM in a linearly separable case [25].

This section presents a brief review of SVMs [8,9,10,11]. Figure 4 shows the approach to identifying the optimal hyperplane ($w^{t}x+b=0)$ with maximum margin for linearly separable classifier in a geometrical view of SVM.

In the linearly separable case, let $\left\{x_{1},\;x_{2},\ldots ,\;x_{z}\right\}$ be the training set and let $y_{i}\in \left\{+1,\;-1\right\}$ be the class label of a *D*-dimensional feature vector $x_{i}$. The margin maximization problem corresponds to [8,9,10,11]:

(2) $$\begin{array}{l} \text{min}\left[\frac{1}{2}w^{T}w+C\sum_{i=1}^{z}\xi_{i}\right]\;\\ s.t.y_{i}\left(w^{T}x_{i}+b\right)\geq 1-\xi_{i};\;\xi_{i}\geq 0;i=1,\;\ldots ,\;z \end{array}$$

Here, $\xi_{i}$ is a penalty for misclassification or classification within the margin, and C(C>0) is a tradeoff parameter between error term and margin. The approach described here for a linear SVM can be extended to the creation of a nonlinear SVM in order to classify linearly inseparable data.

**Figure 5:**
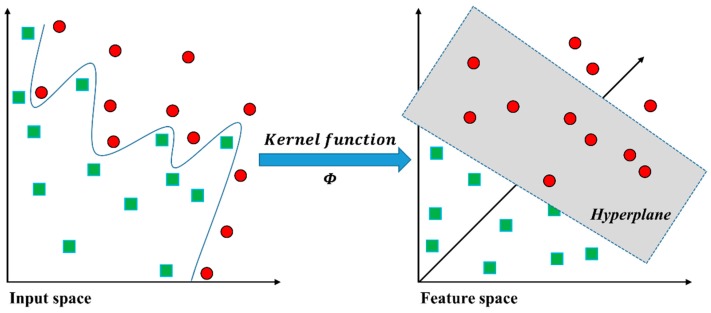
Graphical view of the SVM in the non-linearly separable case [25].

Figure 5 illustrates a solution to the non-linearly separable problem to obtain linear separation by mapping the input training data into the higher-dimensional feature space [8,9,10,11]. In the general mathematical formulation, the kernel function, K, is defined as $K\left(x_{i},\;x_{j}\right)\equiv \phi {\left(x_{i}\right)}^{T}\phi \left(x_{j}\right)$. In particular, the commonly used kernel function is a radial basis function (RBF) as follows:

(3) $$K\left(x_{i},x_{j}\right)=\exp\left(-\gamma {||x_{i}-x_{j}||}^{2}\right),\;\gamma >0$$

Here, γ is a standard deviation parameter [25,26].

## 3. Results

### 3.1. Data Collection

**Figure 6:**
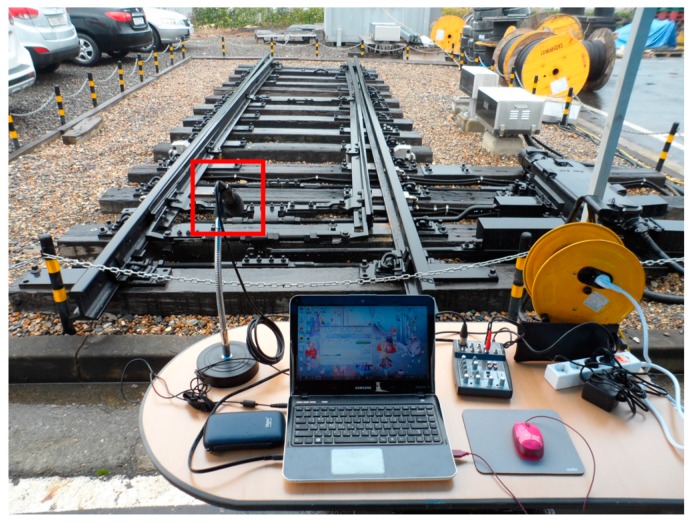
Data collection from NS-AM-type railway points.

**Figure 7:**
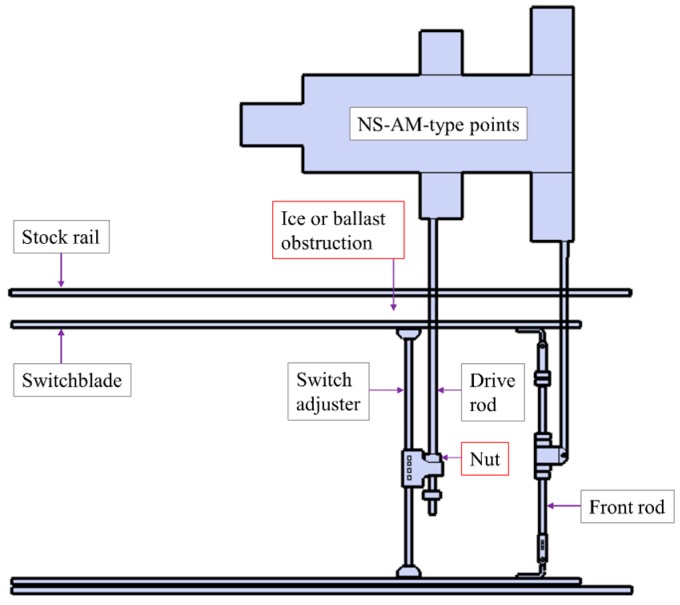
Schematic for NS-AM-type railway points [1].

Audio data were collected from an NS-AM-type railway point machine at Sehwa Company in Daejeon, South Korea, on 1 January 2016. Figure 6 shows a picture of an NS-AM-type railway point machine, which is installed with an audio sensor for this data collection experiment. Figure 7 provides a schematic of the type of NS-AM railway points used in Korea. In general, several types of fault can lead to point failure.

**Figure 8:**
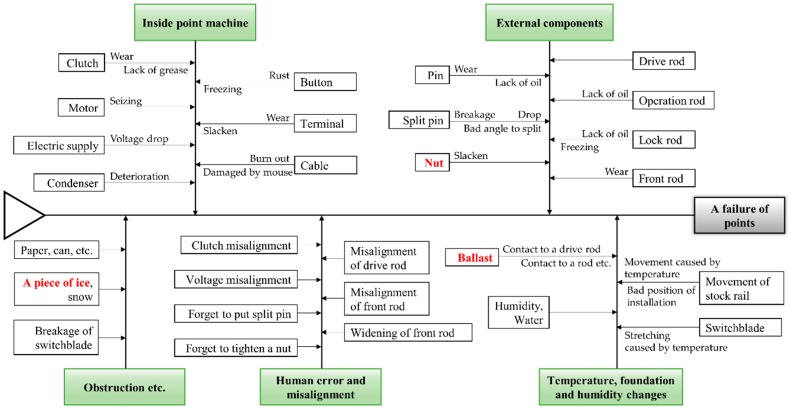
Fishbone diagram of faults for railway point machines [7].

**Figure 9:**
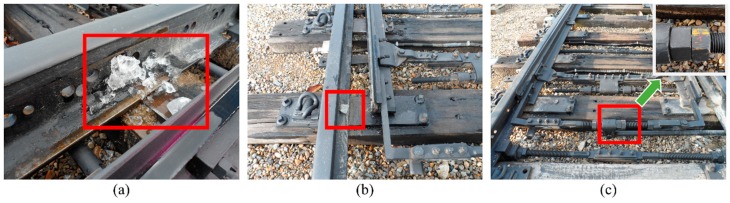
Data collection pictures from simulating three fault conditions: (**a**) ice obstruction; (**b**) ballast obstruction; and (**c**) slackened nut.

Figure 8 shows a fishbone diagram for point failure [7]. As shown in Figure 8, we collected audio data while simulating three fault conditions that include normal data: “ice obstruction”, “ballast obstruction”, and “slackened nut” (see Figure 9). The first two cases concern obstructions between the stock rail and switchblade of the track points. The “slackened nut” scenario may occur when nuts become loose due to a natural process, through train vibration, or maintenance misalignment. Apart from the faults simulated during data collection, to avoid significant faults, a maintenance task was performed before collecting the data.

**Figure 10:**
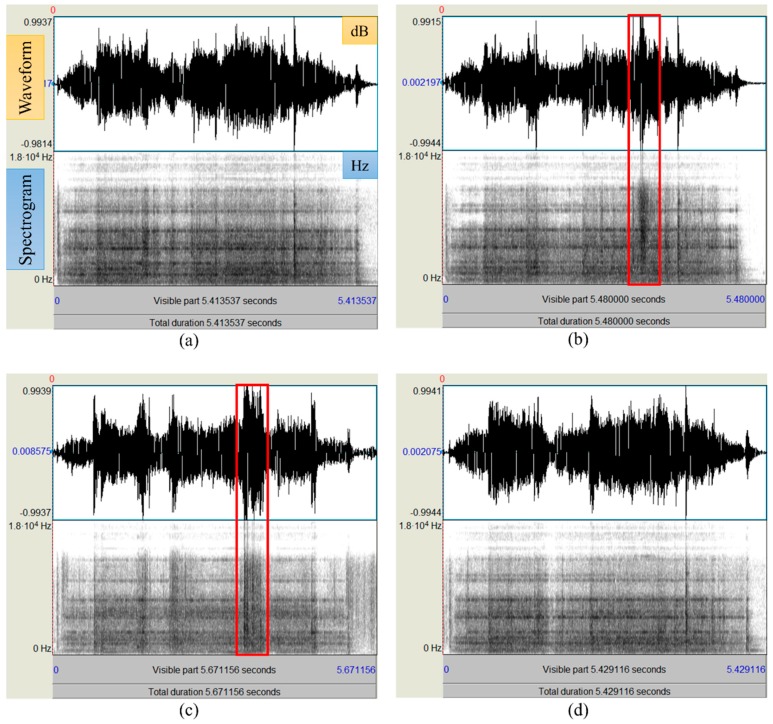
Waveforms and spectrograms for fault and normal sound samples (the rectangle indicates the sound pattern changes at the point of contact between the rod and the obstruction): (**a**) normal; (**b**) ice obstruction; (**c**) ballast obstruction; and (**d**) slackened nut.

The sounds emitted by the railway points were recorded using a SHURE SM137 microphone (Shure Inc., Niles, IL, USA) positioned within one meter of the points (see Figure 6), and recorded onto a Samsung NT-SF310 notebook computer. Adobe Audition 3.0 and R package “tuneR” [17] software were used to digitize the recorded signals in a personal computer with an AC97 soundcard (Realtek, Hsinchu, Taiwan) at 16 bits/44.1 kHz sampling rates. Empirical analysis of the sound spectrogram revealed that ambient (or background) noise existed mainly between 0 and 300 Hz, and that the operational noise of the point machine occurred from 300 to 13,000 Hz. Thus, noise filtering process was performed in a passband of 300–13,000 Hz. Figure 10 illustrates the spectrograms and waveforms of standard and various fault sound models using Praat software (Ver. 6.0.05) [27].

### 3.2. Fault Sound Detection and Classification Results

**Figure 11:**
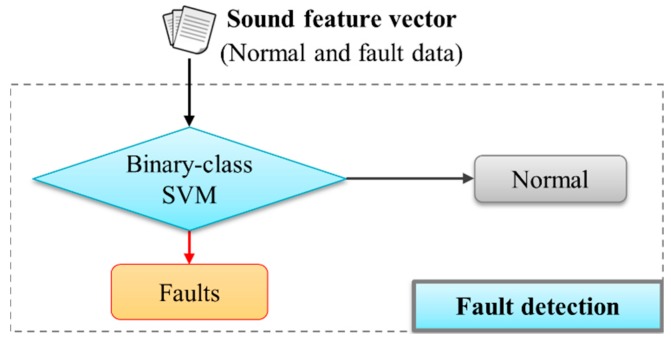
Architecture for fault-sound detection based on a binary SVM.

**Figure 12:**
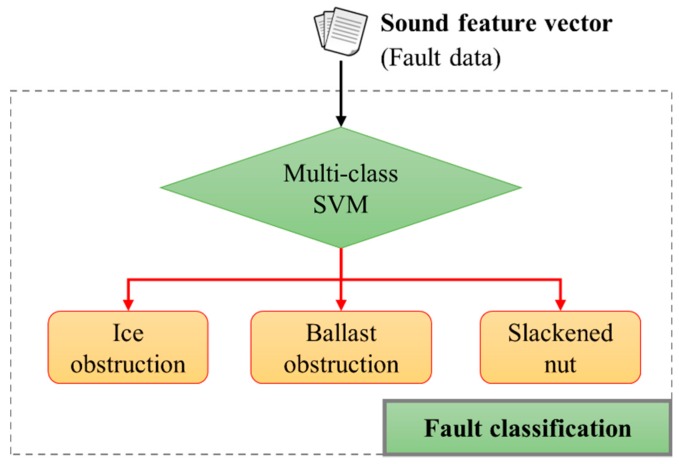
Architecture for fault-sound classification based on a multi-class SVM.

Two different experiments were performed (*i.e.*, one for fault detection with the whole data set, the other for fault classification only using the data labelled as faulty). Figure 11 and Figure 12 show the overall architecture of the SVM-based fault sound detection (a binary-class SVM) and classification system (a multi-class SVM), respectively. The proposed system was implemented using a PC (Intel i7-3770K, 16 GB memory), and the experiments used the Weka [28]. In addition, ten-fold cross-validation with ten repetitions was used. The experiment used 430 fault sound data (140 for “ice obstruction”, 140 for “ballast obstruction”, and 150 for “slackened nut”) and 150 normal sound data. The data set is divided into a training set consisting of half of the original set (randomly chosen), with the other half used as a validation set.

For the MFCC features, 60 frames per sound and 12 cepstral coefficients were used, and 720-dimensional features (12 × 60 = 720) were yielded by using tuneR. The lowest and highest band frequencies were set to 300 and 13,000 Hz respectively, whereas the other parameters were set to default values. In the case of CFS, the dimension of the selected optimal-attribute subsets was reduced to 133 using “CfsSubsetEval” in Weka.

First, an identification test of the proposed mechanism was conducted, to distinguish between fault and normal sounds (see Figure 11). The performance of the proposed system was evaluated via fault detection rate (FDR), false positive rate (FPR), and false negative rate (FNR) [29,30]: True positive (TP: fault sound correctly identified as fault), False positive (FP: normal sound incorrectly identified as fault), True negative (TN: normal sound correctly identified as normal), and False negative (FN: fault sound incorrectly identified as normal):

(4) $$Fault\;Detection\;Rate\;\left(FDR\right)=\frac{TP}{TP+FN}\times 100$$

(5) $$False\;Positive\;Rate\;\left(FPR\right)=\frac{FP}{FP+TN}\times 100$$

(6) $$False\;Negative\;Rate\;\left(FNR\right)=\;\frac{FN}{TP+FN}\times 100$$

A summary of detection results for fault sounds is shown in Table 1. According to the experimental results, when using 720 feature vectors, the fault detection accuracy of the proposed system is 94.1%, and FPR and FNR are 0.6% and 5.9% respectively. Even when only 133 attributes are used, the accuracy is confirmed as satisfactory. We used the corrected resampled *t*-test provided by Weka, with a 95% confidence level, to compare the methods based on the FDR. The results show no significant difference between the 133 and the 720 features. Figure 11 indicates that the detector used in this experiment is a binary SVM. In case of using the entire feature-set, an RBF kernel with 0.0275 gamma was used and C was set at 1.7 for this cross-validation experiment. For CFS, an RBF kernel with 0.061 gamma was used and C was set at 1.91. These values were independently chosen by a GridSearch method in a training phase [28]. Our review of the literature did not identify any previous attempts to detect and classify fault sounds, thus a performance comparison cannot be made.

Secondly, we classified fault sound data into three types: “ice obstruction”, “ballast obstruction”, and “slackened nut” (see Figure 12). In order to measure the classification accuracy of the proposed system, the precision and recall are used as the performance measurements [29,30]:

(7) $$Precision=\frac{TP}{TP+FP}\times 100$$

(8) $$Recall=\frac{TP}{TP+FN}\times 100$$

A summary of the classification results for the studied fault sounds is shown in Table 2. The experimental results show that the precision and recall of the proposed system approach 97.0% when using 720 feature vectors, compared with 93.1% and 93.0% when only 133 features are used. The corrected resampled *t*-test provided by Weka (95% confidence level) was used to compare the methods, showing that precision and recall were significantly better when using the entire features-set than with the 133 features used by CFS. The classifiers were branded as a multi-class SVM (Figure 12). When using the entire feature-set, an RBF kernel with 0.0157 gamma was used and C was set at 1.42. For CFS, an RBF kernel with 0.1073 gamma was used and C was set at 5.45. These values were also independently chosen by a GridSearch method in a training phase.

## 4. Conclusions

The early discovery of anomalies is critical for systems that monitor the condition of railway infrastructure. Failure to uncover faults in a timely and precise manner can become a critical limiting factor in efficiently managing such systems. This work thus presents a timesaving data mining solution for identifying faults through the use of audio data. The railway sound-acquisition process was performed first, while MFCC was isolated from the data-preprocessing segment. Two SVMs were used in the detection and classification of fault sounds, respectively. The experimental results demonstrated cost-effective, automatic detection and diagnosis of railway faults through the analysis of audio data. The combination of MFCC and SVM identified and classified the sounds of railway faults with accuracies of 94.1% and 97.0% respectively. The results confirm that the proposed method provides a credible means of investigating railway sounds for understanding the condition of rail points, whether used alone or in combination with other known methods. Broader testing of the proposed system in commercial production conditions is a purposeful avenue. A complete real-time system is part of our ongoing research.

## Figures and Tables

**Table 1:** Performance of proposed system in fault sound detection.

CFS (133 Features Used)			All Features (720 Used)		
FDR	FPR	FNR	FDR	FPR	FNR
94.3%	2.7%	5.6%	94.1%	0.6%	5.9%

**Table 2:** Performance measurement for fault-sound classification.

Faults	CFS(133 Features Used)		All Features (720 Used)	
	Precision	Recall	Precision	Recall
Ice obstruction	90.8%	88.1%	94.2%	97.3%
Ballast obstruction	91.1%	91.1%	97.2%	93.7%
Slackened nut	97.4%	99.9%	99.7%	100%
**Average**	93.1%	93.0%	97.0% ***^V^***	97.0% ***^V^***

***^V^*** The method is significantly better.
